# Computerized Cognitive Behavioral Therapy Intervention for Depression Among Veterans: Acceptability and Feasibility Study

**DOI:** 10.2196/31835

**Published:** 2022-04-25

**Authors:** Kelly A Stearns-Yoder, Arthur T Ryan, Alexandra A Smith, Jeri E Forster, Sean M Barnes, Lisa A Brenner

**Affiliations:** 1 Veterans Affairs Rocky Mountain Mental Illness Research Education and Clinical Center Rocky Mountain Regional Veterans Affairs Medical Center Aurora, CO United States; 2 Department of Physical Medicine and Rehabilitation University of Colorado Anschutz School of Medicine Aurora, CO United States; 3 Department of Psychiatry University of Colorado Anschutz School of Medicine Aurora, CO United States; 4 Research and Development Service Washington DC Veterans Affairs Medical Center Washington, DC United States; 5 Department of Neurology University of Colorado Anschutz School of Medicine Aurora, CO United States

**Keywords:** computerized cognitive behavioral therapy, depression, veterans, acceptability, feasibility

## Abstract

**Background:**

Computerized cognitive behavioral therapies (cCBTs) have been developed to deliver efficient, evidence-based treatment for depression and other mental health conditions. Beating the Blues (BtB) is one of the most empirically supported cCBTs for depression. The previous trial of BtB with veterans included regular guidance by health care personnel, which increased the complexity and cost of the intervention.

**Objective:**

This study, conducted by researchers at a Veterans Affairs Medical Center, aims to test the acceptability and feasibility of unguided cCBT for depression among US military veterans.

**Methods:**

To examine the acceptability of BtB delivered without additional peer or other mental health care provider support, a before-and-after trial was conducted among United States (US) military veterans experiencing mild to moderate depressive symptoms. The feasibility of the study design for a future efficacy trial was also evaluated.

**Results:**

In total, 49 veterans completed preintervention assessments and received access to BtB, and 29 participants completed all postintervention assessments. The predetermined acceptability criterion for the intervention was met. Although the predetermined feasibility criteria regarding screening eligibility rate, number of BtB modules completed, and completion of a posttreatment assessment were not met, the results were comparable with those of other cCBT studies.

**Conclusions:**

This is the first study among US military veterans to demonstrate support for the implementation of cCBT for depression without the assistance of a mental health professional or a peer support specialist, suggesting that stand-alone computer-aided interventions may be viable. Ideas for improving feasibility in future trials based on this study are discussed.

## Introduction

### Background

Depressive disorders are among the most common mental health conditions associated with high morbidity [1]. Compared with the general population, US military veterans are at an increased risk of depression [2]. Approximately 1 in 3 veterans visiting primary care clinics has some symptoms of depression, 1 in 5 has serious symptoms that indicate the need for further evaluation, and 1 in 9 requires psychotherapy or antidepressant treatment for major depression [3].

Although depressive disorders are common and undertreated conditions [4-6], there are effective treatments, including cognitive behavioral therapy (CBT) and pharmacotherapy [7]. Research supports the notion that patients generally prefer psychological therapy to medication [8,9]. However, there is a critical need for trained mental health professionals to treat depressive disorders among individuals in the US, including the US veterans [10]. Although CBT training programs are available for providers within the Department of Veterans Affairs (VA), additional factors can limit individuals’ ability to engage in traditional face-to-face therapy, including the resources required to travel to the clinic for repeated appointments, which may not be available at the most convenient time [11]. Alternative methods have been developed to address limitations inherent to traditionally delivered psychotherapy and facilitate efficient, evidence-based, and appropriate treatment [12]. An alternative method for providing evidence-based psychotherapy to a larger population is the use of computer-aided therapies.

In the past 40 years, efficacious computerized cognitive behavioral therapies (cCBTs) have been developed to treat depression and other mental health conditions. Most cCBT programs can be accessed using the internet (eg, from an individual’s home or a public library) and are highly interactive (eg, audio, video, and animations) [13]. cCBTs have the potential to address some accessibility concerns related to mental health care, as they can be administered at home during times that are most convenient for the patient, thereby reducing barriers to care, including those posed by geography, transportation costs, travel time, and childcare. Because cCBTs generally require less provider time, they can also be cost-effective alternatives to traditional face-to-face psychotherapy.

Meta-analyses have found that cCBT for depression and anxiety disorders is similarly effective to traditional face-to-face CBT in reducing symptoms of depression and anxiety [14-16]. Posttreatment satisfaction with cCBT in the general population has also been studied [17,18] and is similar to that of face-to-face therapies [19,20]. Patients receiving cCBT reported being as satisfied as those receiving CBT from a clinician [21] and more satisfied than those receiving treatment as usual [22,23]. These findings suggest that cCBT is a viable alternative for treating common mental health symptoms.

One of the most empirically supported cCBTs for depression is Beating the Blues (BtB), an 8-session, self-administered, and interactive cCBT program (see the *Methods* section). Although BtB is self-administered, the program offers a provider portal to track patient progress and facilitate health care provider or peer support to encourage treatment engagement. Participation in the BtB program has been associated with reductions in mild to moderate symptoms of depression and anxiety [24-26], and it has been recommended as the gold standard for treating mild to moderate symptoms of depression in primary care contexts [13,16,27]. In randomized controlled trials, BtB has also been established as a cost-effective intervention [16,28]. In addition, BtB has demonstrated effectiveness in addressing depression in older adults [29].

A recent study has demonstrated the acceptability and feasibility of BtB with peer support for treating mild to moderate symptoms of depression in veterans receiving primary care or outpatient mental health services [30]. However, because the study intervention included an additional component of weekly interactions with a veteran peer support specialist, it is unclear whether the effects observed were associated with the BtB program, the peer support specialist, or the combination.

Some studies have shown that veterans respond differently to evidence-based psychotherapies, including CBT for depression [31]. In terms of variables associated with treatment response, including psychiatric comorbidity and lack of stable housing, Veterans receiving health care from the VA differ from other US cohorts [32]. For example, comorbid posttraumatic stress disorder, which is more common in veterans than civilians, may reduce the effectiveness of depression treatments [31].

### Objectives

This study aims to examine the acceptability and feasibility of delivering BtB without additional peer or mental health care provider support among veterans receiving care at a Veterans Affairs Medical Center (VAMC). Acceptability refers to the suitability of an intervention from the perspective of participants [33]. Feasibility refers to the goodness of fit between an intervention and the system in which it is disseminated. Aligned with the guidance provided by Areán and Kraemer [34], our feasibility assessment focused on the ease of implementation of study design elements, which were participant recruitment, enrollment, and retention. Before commencing the study, a set of a priori criteria (see the *Acceptability and Feasibility Criteria and Analysis* subsection in the *Methods* section) was established to measure the acceptability and feasibility per best practices in pilot study designs [35].

## Methods

### Design

All participants were allocated to receive the BtB intervention following a before-and-after trial design [36]. This study was approved by the Colorado Multiple Institutional Review Board and the local VA Research and Development Committee. A Health Insurance Portability and Accountability Act waiver was granted for the entire study, as all procedures were completed remotely (over the phone with the participant or on the web via a survey link). Data collection occurred between September 2019 and February 2020. After a research team member read the consent information sheet to the potential participants, verbal consent was obtained. Participants could also download a copy of the consent form via the Research Electronic Data Capture (REDCap; Vanderbilt University) [37] platform, a web-based portal for participants to complete self-report study measures. Following verbal consent, the interviewer conducted a telephone eligibility screening. If the participant was eligible for the study, the interviewer ended the telephone conversation and sent a link to a web-based survey of preintervention (baseline) measures to be completed on the REDCap platform. After completing the preintervention measures, participants were provided a link to register for and access the BtB web-based intervention. All the BtB content, including an introductory session and 8 content modules, could be completed autonomously via an internet browser interface without facilitation by a health care provider, peer, or study staff. Although the core content of BtB can be completed in as few as 8 weeks, participants were given access to the program for 12 weeks. After completing 8 modules or at the end of the 12 weeks, participants were emailed a second link to complete postintervention assessments via REDCap. All participants were compensated for completing the pre- and postintervention surveys.

### Participants

Initially, veteran participants were recruited exclusively via referral from a Primary Care and Mental Health Integration (PC-MHI) psychologist. PC-MHI psychologists are embedded in VA Patient Aligned Care Teams within primary care clinics to provide brief mental health assessments, referrals, and interventions. The study team received 45 referrals from the PC-MHI psychologist, and 29 veterans consented to participate in the study. Of the 29 veterans, 18 (62%) did not complete any BtB modules, 4 (14%) were lost to follow-up, and 7 (24%) completed postintervention surveys. Because the study did not meet its targets for recruitment, enrollment, and data collection, the study team implemented a new recruitment strategy that involved identifying potential participants using electronic medical records (EMRs). This paper does not report data from participants recruited via PC-MHI psychologist referral because they might systematically differ from those recruited using EMRs. In addition, we may use the EMR recruitment method in future clinical trials based on this feasibility and acceptability study.

For recruitment using EMRs, potential participants were identified from the VA Corporate Data Warehouse. The records were electronically searched for veterans (1) eligible to receive care at a particular VAMC between August 2017 and July 2019 who (2) were administered the Patient Health Questionnaire-9 (PHQ-9), a brief self-report measure of depression and (3) received a total score between 5 and 15 points, which indicates the presence of mild to moderate symptoms of depression [38]. Considering previous studies supporting the efficacy of cCBT in the treatment of depression and the statistical relationship between depression and suicidal behavior among veterans [39], we chose to recruit veterans based on symptoms of depression instead of other symptomatology. Veterans meeting these criteria were sent a letter inviting them to participate in the study. The letter briefly described the study and instructed interested veterans to call a research team member to be screened for eligibility.

During the telephone screening, potential participants self-reported whether they met the following inclusion criteria: (1) being a US military veteran aged between 18 and 89 years; (2) having a score of 5 to 15 on a verbally administered PHQ-9, which determined whether the veteran currently endorsed mild to moderate depressive symptoms [38]; (3) able to write, read, and speak English; and (4) having reliable access to the internet. The following exclusion criteria were also assessed: (1) inability to complete the assessment sessions or participate in the intervention because of visual or hearing impairment, severe psychiatric symptoms (eg, active psychosis or imminent suicide risk), or severe cognitive impairment; (2) membership in a vulnerable population (eg, pregnant women and prisoners); and (3) self-reported current participation in another mental health intervention study. The telephone-administered PHQ-9 was only used for screening purposes; another PHQ-9 was administered electronically via REDCap along with the other preintervention assessments to collect the data used in the study analyses.

### Measures

The measures administered during pre- and postintervention are shown in Multimedia Appendix 1; Table S1.

#### Client Satisfaction Questionnaire

The Client Satisfaction Questionnaire (CSQ) [40] is an 8-item questionnaire used to assess participants’ satisfaction with an intervention. Scores range from 8 to 32, with higher scores indicating greater satisfaction. A score of 24 or higher indicates that the average item rating was in the *mostly satisfied* or better range. The CSQ has good reliability and validity and has been frequently used to evaluate mental health care [41]. The CSQ was administered during the postintervention assessment and was a measure of acceptability.

#### Generalized Anxiety Disorder Inventory-7

The Generalized Anxiety Disorder Inventory-7 (GAD-7) [41] is a 7-item self-report measure that assesses symptoms related to generalized anxiety disorder. Participants respond to items using a 4-point Likert scale ranging from 0 to 3 (*not at all* to *nearly every day*, respectively). Participants endorse the items based on how they felt in the last 2 weeks. Higher scores reflect greater symptoms of generalized anxiety. The GAD-7 has demonstrated good reliability and validity [41]. As our trial was specifically designed to examine BtB acceptability and feasibility and not as an efficacy trial (eg, it did not include a control condition), only baseline GAD-7 scores are reported in this paper.

#### Internet Evaluation and Utility Questionnaire

The Internet Evaluation and Utility Questionnaire (IEUQ) [42] is a 13-item measure that examines the participants’ experiences of a web-based intervention. The IEUQ was adapted for this study to refer specifically to BtB. The constructs measured included items on ease of use, convenience, engagement, enjoyment, layout, privacy, and overall satisfaction. The IEUQ was administered to gather further information on the participants’ impressions of the aspects of the intervention that were relevant to its acceptability (eg, convenience). Participants responded to items on a 5-point Likert scale from 0 (*not at all*) to 4 (*very*) or rated them as *not applicable*. Previous research indicates that the IEUQ has adequate reliability [42,43].

#### Internet Impact and Effectiveness Questionnaire

The Internet Impact and Effectiveness Questionnaire (IIEQ) [42] is a 20-item instrument that measures individuals’ perceptions of the effectiveness of a web-based intervention. The perceived impact is measured in terms of helpfulness, knowledge gains, treatment effectiveness for self and others, long-term effectiveness, quality of life, mood, physical activity, family and peer relationships, social activity, school or work attendance and performance, treatment implementation, goal orientation, confidence in the ability to manage conditions, relapse prevention, and service reduction. The perceived or actual effectiveness of the intervention is likely to influence patients’ perceptions of acceptability [44]. This measure was adapted for this study to refer specifically to BtB. Participants responded to items using a 5-point Likert scale ranging from 0 (*not at all*) to 4 (*very*). There was also a *not applicable* option. Adequate psychometric properties have been demonstrated previously [42,43].

#### PHQ-9 Scores

The PHQ-9 is a 9-item measure that assesses symptoms of depression. Participants respond to items using a 4-point Likert scale ranging from 0 to 3 (*not at all* to *nearly every day*, respectively), endorsing them based on how they felt in the last 2 weeks. Higher scores reflect greater symptoms of depression. The PHQ-9 has demonstrated good psychometric properties [38]. Similar to the GAD-7, only baseline PHQ-9 scores are reported in this paper.

#### Reasons for Termination–Adapted

The reasons for termination (RFT) scale [45] assesses 10 common reasons why patients terminate therapy and the impact these reasons have on termination. Thus, the RFT scale gathers information about the elements of a therapy that reduce its acceptability. If a participant completed between 1 and 7 modules of the BtB treatment within 12 weeks of the initial log-in to the BtB treatment website (ie, began but did not finish the therapy within the allotted time), this measure was included with the postintervention measures collected via a REDCap survey link.

### Intervention

BtB is a computerized cognitive behavioral intervention program aimed at reducing depressive and anxiety symptoms [23]. BtB was designed explicitly to implement the standard CBT model of depression and anxiety and include the therapeutic elements that make up traditional CBT psychotherapy (eg, thought records [46]). The web-based intervention consisted of a 15-minute introductory session followed by 8 interactive modules, usually taken weekly. Each weekly module lasted approximately 50 minutes, with homework projects to complete between the modules (eg, problem diaries, thought records, and behavioral experiments).

### Acceptability and Feasibility Criteria and Analysis

The a priori criterion for assessing the acceptability of the intervention was that ≥70% of the participants had a score of ≥24 on the CSQ. A score of ≥24 was selected as the cutoff because it indicates that the average item rating was in the *mostly satisfied* or better range. Additional acceptability data were collected from the IIEQ, IEUQ, and RFT. The a priori criteria for assessing the feasibility of the study were (1) ≥70% of potential participants would meet the eligibility criteria assessed during the telephone screening, (2) ≥70% of those found eligible would consent to participate in the study, (3) ≥60% of those who completed the preintervention survey would complete all the intervention modules, and (4) ≥75% of those who registered for BtB and completed at least one module would complete the postintervention survey measures.

### Study Procedures

**Figure 1:**
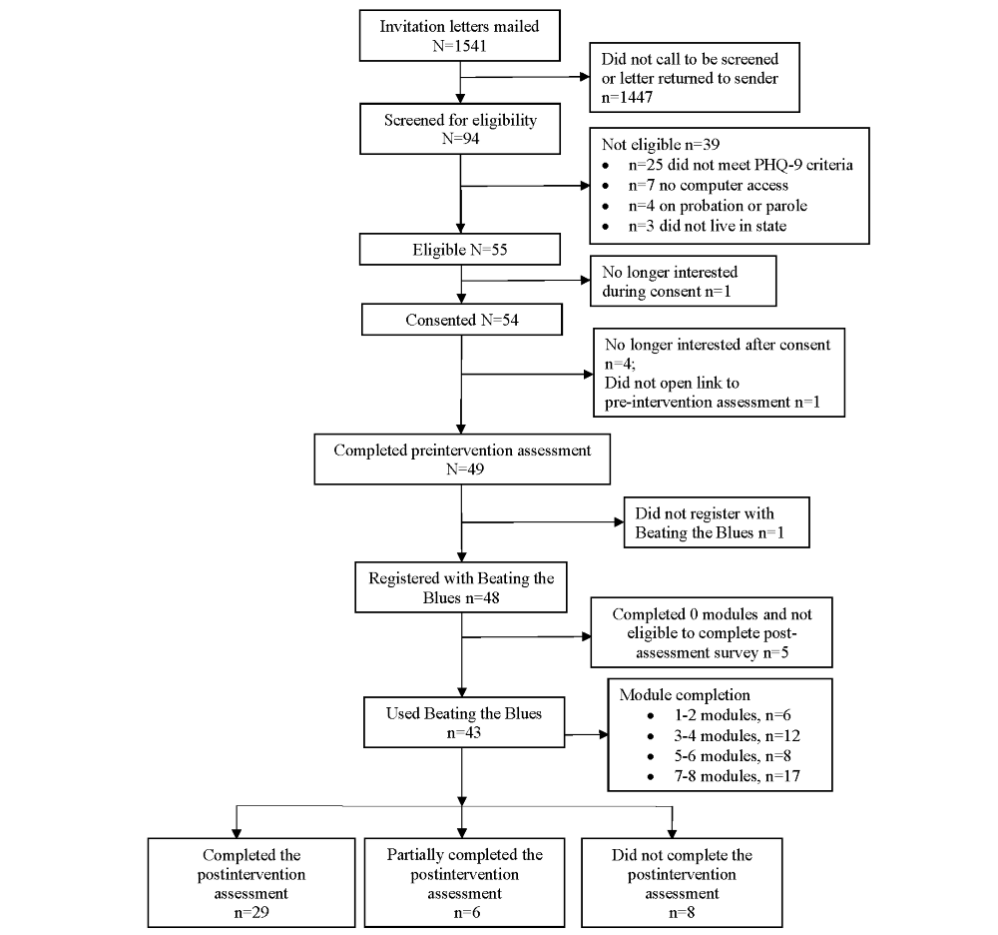
Enrollment flow diagram. PHQ-9: Patient Health Questionnaire-9.

Study recruitment, enrollment, and data collection were conducted at multiple time points (Figure 1).

## Results

### Baseline Demographics

Of the 54 veterans recruited via EMRs who consented to participate in the study, 49 (91%) used the emailed link to complete the baseline assessment measures. The sample was predominantly male (43/49, 88%) and White (37/49, 76%). At baseline, most participants reported moderate depression (ie, a PHQ-9 score in the moderate depression range of 10-14) and mild anxiety (ie, a GAD-7 score in the mild anxiety score range of 5-9). Further details regarding demographic characteristics are shown in Table 1.

**Table 1:** Baseline demographics (N=49).

Variable			Value
Age (years), mean (SD); range			57.1 (9.9); 35-74
**Self-identified gender, n (%)**			
	Male	43 (88)	
	Female	6 (12)	
**Racial background, n (%)**			
	White	37 (76)	
	Black or African American	7 (14)	
	Native American or Alaskan	3 (6)	
	Asian	1 (2)	
	Other	1 (2)	
**Ethnicity, n (%)**			
	Hispanic or Latinx	7 (14)	
	Non-Hispanic or Non-Latinx	41 (84)	
	Refused to respond	1 (2)	
**Highest level of education, n (%)**			
	High-school diploma or equivalent	4 (8)	
	Some college, no degree	18 (37)	
	Associate’s degree	7 (14)	
	Bachelor’s degree	9 (18)	
	Master’s degree	8 (16)	
	Doctoral degree	3 (6)	
**Marital or relationship status, n (%)**			
	Married	28 (57)	
	Single	10 (20)	
	Cohabiting	1 (2)	
	Divorced or separated	10 (20)	
**Sexual orientation, n (%)**			
	Heterosexual	49 (100)	
**Employment status, n (%)**			
	Employed full-time	13 (27)	
	Employed part-time	4 (8)	
	Unemployed, not currently seeking employment	8 (16)	
	Unemployed, seeking employment	1 (2)	
	Retired	22 (45)	
	Refused to respond	1 (2)	
Currently a student, n (%)			2 (4)
Currently homeless, n (%)			0 (0)
Ever homeless, n (%)			16 (30)
**Branch of military service, n (%)**			
	Army—active duty	21 (43)	
	Army reserve	5 (10)	
	Army national guard	3 (6)	
	Air force—active duty	13 (27)	
	Air force reserve	7 (14)	
	Air national guard	5 (10)	
	Navy—active duty	8 (16)	
	Navy reserve	3 (6)	
	Marine corps—active duty	7 (14)	
	Marine corps reserve	2 (4)	
	Coast guard—active duty	1 (2)	
Total months of active duty service, mean (SD)			78.5 (73.4)
Total months of reserve service, mean (SD)			24.4 (54.9)
**Service era, n (%)**			
	Vietnam (August 1964-May 1975)	13 (27)	
	Post-Vietnam/Peacetime (May 1975-July 1990)	24 (49)	
	Desert-Storm/Desert-Shield (August 1990-Aug 2001)	22 (45)	
	OEF^a^/OIF^b^/OND^c^ (September 2001-Present)	14 (29)	
	Other	3 (6)	
**Highest rank at separation or current rank, n (%)**			
	Enlisted	35 (71)	
	Noncommissioned officer	10 (20)	
	Officer	4 (8)	
Number of deployments, mean (SD)			2.2 (3.3)
Number of combat tours, mean (SD)			0.9 (1.2)
PHQ-9^d^ at baseline, mean (SD)			12.1 (4.7)
GAD-7^e^ at baseline, mean (SD)			9.0 (4.8)

^a^OEF: Operation Enduring Freedom.

^b^OIF: Operation Iraqi Freedom.

^c^OND: Operation New Dawn.

^d^PHQ-9: Patient Health Questionnaire-9.

^e^GAD-7: Generalized Anxiety Disorder Inventory-7.

### Acceptability

The CSQ was used to examine whether participants found the BtB intervention acceptable, which was operationalized as a CSQ score of ≥24. Among the 35 participants who completed the CSQ, 26 (74%; 95% CI 57%-88%) received a score of ≥24, which met the a priori criterion of ≥70% (CSQ mean 25.2, SD 4.8).

Acceptability data were also collected using the IEUQ, IIEQ, and RFT. The responses to the IEUQ are presented in Table 2. On the IEUQ, many participants reported liking BtB and being satisfied with the intervention. Data from the IEUQ suggested that BtB was *mostly* or *very easy* to use (23/30, 77%) and generally kept participants’ attention. On the IEUQ, most of the participants (21/30, 70%) found that the internet was a *mostly* or *very good* method for delivering CBT. In addition, participants (18/30, 60%) reported they were *mostly* or *very likely* to come back to BtB if depression difficulties continued or returned.

Responses to the IIEQ regarding the perceived effectiveness of BtB are shown in Table 3. On the IIEQ, most participants reported that BtB *somewhat* or *mostly* improved depressive symptoms (18/29, 62%), quality of life (15/29, 51%), and mood (15/29, 51%). Participants felt they *somewhat* or *mostly* gained more knowledge while using BtB (21/29, 72%), and they were *somewhat* or *mostly* prepared to handle depressive symptoms in the future (16/29, 55%). In addition, they were *mostly* or *very likely* to recommend BtB to others with similar problems (20/29, 69%).

Although many participants found the intervention acceptable, some did not complete all the 8 modules of the intervention (Figure 1). The RFT questionnaire was administered to participants who completed between 1 and 7 modules (Table 4). Some participants reported that the barriers to completing all modules included practical problems (3/14, 21%), time constraints (5/14, 36%), and medical reasons (4/14, 29%). None of the participants who completed the RFT reported that they chose to stop the intervention because they were dissatisfied or wanted a different intervention.

**Table 2:** Responses to the Internet Evaluation and Utility Questionnaire after the assessment (n=30).

Domain	Response, n (%)					
	Not at all	Slightly	Somewhat	Mostly	Very	Not applicable or no response
Easy to use	0 (0)	2 (7)	3 (10)	9 (30)	14 (47)	2 (7)
Convenient to use	0 (0)	1 (3)	3 (10)	8 (27)	14 (47)	4 (13)
Keeps interest and attention	0 (0)	1 (3)	7 (23)	17 (57)	3 (10)	2 (7)
Liked BtB^a^	1 (3)	2 (7)	4 (13)	10 (33)	10 (33)	3 (10)
Liked how BtB looked	0 (0)	1 (3)	4 (13)	9 (31)	9 (31)	7 (23)
Worried about privacy	17 (57)	5 (17)	4 (13)	2 (7)	1 (3)	1 (3)
Satisfied	2 (7)	2 (7)	4 (13)	11 (37)	9 (30)	20 (7)
Good fit	1 (3)	2 (7)	8 (27)	8 (27)	9 (30)	2 (7)
Usefulness	0 (0)	2 (7)	3 (10)	8 (27)	13 (43)	4 (13)
Easy to understand	0 (0)	0 (0)	5 (17)	3 (10)	19 (63)	3 (10)
Trust the information	0 (0)	0 (0)	2 (7)	11 (37)	13 (43)	4 (13)
Likely to come back	2 (7)	1 (3)	7 (23)	7 (23)	11 (37)	2 (7)
Liked delivery via internet	1 (3)	3 (10)	3 (10)	4 (13)	17 (57)	2 (7)

^a^BtB: Beating the Blues.

**Table 3:** Responses to the Internet Impact and Effectiveness Questionnaire after the assessment (n=29).

Domain	Response^a^, n (%)					
	Not at all	Slightly	Somewhat	Mostly	Very	Not applicable or no response
Improved depressive symptoms	1 (3)	6 (21)	10 (34)	8 (28)	3 (10)	1 (3)
More knowledge	0 (0)	4 (14)	9 (31)	12 (41)	2 (7)	2 (7)
How well it worked	1 (3)	5 (17)	9 (31)	9 (31)	5 (17)	0 (0)
How well it can work for others	0 (0)	2 (7)	6 (21)	7 (24)	6 (21)	8 (28)
Work as long-term cure	2 (7)	3 (10)	11 (38)	7 (24)	4 (14)	2 (7)
Improved quality of life	1 (3)	7 (24)	12 (41)	3 (10)	3 (10)	3 (10)
Improved mood	1 (3)	7 (24)	12 (41)	3 (10)	3 (10)	3 (10)
Improved physical activities	5 (17)	9 (31)	7 (24)	5 (17)	1 (3)	2 (7)
Improved relationships with family	3 (10)	9 (31)	5 (17)	2 (7)	4 (14)	6 (21)
Improved relationships with friends, peers, or coworkers	1 (3)	11 (38)	8 (28)	3 (10)	1 (3)	5 (17)
Improved social life, such as visiting friends and engaging in community activities	5 (17)	7 (24)	8 (28)	5 (17)	1 (3)	3 (10)
Improved school or work attendance	4 (14)	5 (17)	0 (0)	1 (3)	2 (7)	17 (59)
Improve school or work performance	4 (14)	7 (24)	0 (0)	1 (3)	2 (7)	15 (52)
Able to follow through with BtB^b^ recommendations	1 (3)	7 (24)	5 (17)	10 (34)	5 (17)	1 (3)
Able to reach goals at beginning of BtB	3 (10)	6 (21)	10 (34)	7 (24)	2 (7)	1 (3)
Help feel more confident to manage depressive symptoms	2 (7)	4 (14)	11 (38)	6 (21)	4 (14)	2 (7)
Likely to recommend BtB to others with similar problems	1 (3)	2 (7)	4 (14)	7 (24)	13 (45)	2 (7)
Prepared to handle depressive symptoms in future	1 (3)	6 (21)	7 (24)	9 (31)	5 (14)	2 (7)
Reduce the number of office visits with a health professional	8 (28)	7 (24)	2 (7)	3 (10)	3 (10)	6 (21)
Reduce the number of phone calls and emails with a health professional	5 (17)	3 (10)	4 (14)	2 (7)	4 (14)	11 (38)

^a^A participant completed the Internet Evaluation and Utility Questionnaire but stopped filling out the postintervention assessments before completing the Internet Impact and Effectiveness Questionnaire.

^b^BtB: Beating the Blues.

**Table 4:** Responses to the reasons for termination questionnaire at the postintervention assessment (n=14).

Domain	Response, n (%)		
	Yes	No	Not applicable or no response
Practical problems	3 (21)	10 (71)	1 (7)
Time problems	5 (36)	8 (57)	1 (7)
Medical reasons	4 (29)	9 (64)	1 (7)
Problems improved and no longer felt a need for BtB^a^	0 (0)	12 (86)	2 (14)
Not improving as much as wanted to	2 (14)	10 (71)	2 (14)
Dissatisfied with BtB	0 (0)	11 (79)	3 (21)
Wanted a different intervention	0 (0)	10 (71)	4 (29)
Pressured or advised by others (eg, friends, spouse, or other people who criticized participation in BtB or said they did not need it)	0 (0)	13 (93)	1 (7)
Afraid that employer or others would find out about participation in BtB	0 (0)	13 (93)	1 (7)
Other reasons that led to ending participation	6 (43)	6 (43)	2 (14)

^a^BtB: Beating the Blues.

### Feasibility

Of the 94 veterans identified via EMRs and screened, 59% (55/94) were eligible for the study, which did not meet the a priori feasibility criterion of ≥70%. Of those eligible, 98% (54/55) consented to participate in the study (Figure 1), meeting the a priori feasibility criterion of ≥70%, and 49 (91%) of them used the emailed link to complete the preintervention assessments. A participant did not register with the BtB program after completing the preintervention assessment, leaving 48 participants in the study. Of these, 33% (16/48) completed all 8 BtB modules, which did not meet the a priori feasibility criterion of ≥60%. Among the other registered participants, 19% (9/48) completed 5 to 7 modules, 38% (18/48) completed 1 to 4 modules, and 10% (5/48) did not complete any module after registering for BtB. Participants were not eligible to complete the postintervention survey if they did not register with BtB and complete at least one module. Of the 43 participants who completed at least one BtB module, 67% (29/43) completed all postintervention measures, which did not meet the a priori criterion of ≥75%, and 6 (14%) individuals partially completed the postintervention survey, meaning that 81% (35/43) of participants completed at least part of it. Of note, based on demographics and preintervention measures, participants who completed this survey did not differ significantly from those who did not (Multimedia Appendix 1; Table S2).

## Discussion

### Principal Findings

The results of this pilot study indicate that the a priori acceptability criterion for the intervention was met. Veterans generally found BtB helpful and easy to use. Most of the veterans who completed the postintervention assessment reported that they were able to complete the tasks associated with the intervention, that they found the intervention helpful, and that they would recommend it to other veterans with similar problems. The observed module completion rate was similar to the rates found with other unguided cCBT interventions [47] and with BtB in non-Veterans [48]. The completion rate was also similar to that found in a previous study of a peer-supported implementation of BtB in veterans [30]. Among the veterans who completed the RFT measure, the most common reasons for treatment discontinuation were related to external factors (eg, time constraints). None of the veterans reported discontinuing because of dissatisfaction with the treatment.

Concerning feasibility, three of four a priori criteria were not satisfied. We believe that a reason for this outcome is that, in retrospect, the criteria selected before the pilot study were overly conservative. In this study, participant recruitment via EMRs occurred rapidly (within 2 months), which did not allow for larger modifications to aspects of the design (eg, the retention plan) while the study was being conducted. However, in line with the advice of Thabane et al [35], conducting this pilot study has suggested several modifications to the study design that we will implement in future iterations of this research protocol.

One of the initial feasibility criteria was that ≥70% of veterans referred to the study would meet the secondary screening criteria, which included current mild to moderate depressive symptoms. As depression is a waxing and waning condition [49], it is unsurprising that many veterans’ symptoms improved or worsened between the initial administration of the PHQ-9 recorded in the EMR and when they were screened for this study. Furthermore, undertaking this pilot trial suggested that only modest experimenter and participant efforts were required to conduct the telephone screening to confirm the presence of current mild to moderate symptoms of depression. Given these considerations, we concluded that the 59% eligibility rate among veterans who received a telephone screening was more than sufficient to demonstrate the feasibility of enrolling this population in future larger efficacy trials. Additional feasibility-related modifications of the study protocol to increase the percentage of veterans with eligible PHQ-9 scores at phone screening (eg, requiring a more recent PHQ-9 administration in the EMR) would not be required.

Another a priori feasibility criterion was that ≥60% of veterans would complete all the 8 modules associated with the treatment. In retrospect, both the percentage of veterans required to complete the modules and the number of modules required by this criterion were overly conservative. Furthermore, we believe that easily implemented changes to the protocol (described below) may further increase the mean number of modules completed. Regarding the number of modules required, a more appropriate number might have been derived from the previous study of BtB in veterans [30], which found that those who completed 5 or more modules of BtB showed statistically significant improvements in symptoms. In our sample, 52% (25/48) of veterans completed 5 or more modules, compared with only 33% (16/48) who completed all 8 modules. Although a higher percentage of veterans met the 5 or more module completion rate criteria, the fact that this 52% completion rate is still less than the 60% rate that we targeted suggests that we should consider modifications to the study protocol to increase the proportion of veterans who complete at least five modules. Of relevance to identifying potentially useful protocol modifications, a previous study found that simple weekly email reminders to complete web-based psychotherapy modules increased the mean number of modules completed by 50% (from 3.7 modules to 5.5 modules) [50]. Researchers have obtained further increases in the efficacy of such reminder emails by adjusting their content (eg, sending emails that inform the user of new content rather than simply reminding them to complete a session) and timing (eg, emailing users after 2 weeks of absence rather than longer periods) [51]. In future iterations of this protocol, we will implement such email reminders to improve the mean number of modules completed. It should be noted that only 40% of veterans completed at least five modules in the previous trial of BtB that used peer support specialists to facilitate the completion of BtB modules [30] compared with 52% (25/48) in our unfacilitated trial, suggesting that the greater investment of resources required to include peer support specialists in the intervention may not necessarily increase the average number of modules completed by participants.

Regarding the criterion for the proportion of participants who needed to complete the required number of modules, we believe that setting this criterion to ≥60% of participants was also too conservative. A core advantage of BtB (and other cCBT interventions) is that it requires less time and resources to be administered to an individual, both from the perspective of veterans and the health care system. Therefore, whereas traditionally implemented treatments might indeed require a low noncompletion rate to justify their research or clinical use, a low-intensity intervention such as BtB probably may not. To reiterate, we believe that the study protocol should be modified to increase the mean number of modules completed. However, we also conclude that a lower minimum completion rate might have been a more useful feasibility criterion given the low veteran and provider burden associated with BtB.

The final a priori feasibility criterion that was not met required that ≥75% of veterans complete the postintervention assessment. In reflecting upon our pilot study, we believe that some small alternations to the experimental design could have improved the observed postintervention survey completion rate of 67%. Previous studies on methods to increase participant retention and assessment completion suggest that using one or more of the following strategies might have increased the rates of postintervention survey completion: a telephone follow-up to the assessment invitation; shortened versions of questionnaires where possible; and, reminders to nonrespondents [52]. We also note that the literature on methods to improve participant retention is growing, and efforts to systematically identify efficacious retention strategies are underway [53].

To our knowledge, this study is the first to demonstrate that a substantial percentage of veterans can complete BtB without the assistance of a mental health professional or peer support specialist [30]. This is important because one of the main virtues of cCBT interventions is the possibility of providing evidence-based CBT interventions to a larger number of individuals [54]. A strength of our study was its broad eligibility and few exclusion criteria, which allowed us to recruit a veteran sample that was relatively representative of the broader population of veterans to whom a BtB intervention might be provided in regular clinical practice (eg, veterans receiving care at a VAMC and reporting mild to moderate symptoms of depression).

### Limitations

Our study has several important limitations. First, the relatively small number of veteran participants who were women, non-White, or Hispanic did not represent the entire US veteran population [55] and limited the ability to demonstrate the acceptability of BtB in these important populations. However, other studies have generally shown that cCBT interventions, including BtB, have similar acceptability among women and members of minority groups [56]. Second, veterans with more severe depression (scores >15 on the PHQ-9) were excluded from our study, limiting our ability to draw conclusions regarding the feasibility of the design and acceptability of the intervention among veterans with more severe symptoms of depression. However, this exclusion criterion is consistent with the practice of health care systems where cCBT has already been successfully implemented (eg, in the United Kingdom [57]). In these systems, cCBT (and other less resource-intensive interventions) are initially provided to a larger population of individuals with mild to moderate depression symptoms, whereas a smaller population of high-acuity patients receive more resource-intensive interventions, such as in-person psychotherapy [58]. Finally, 17% (8/48) of the participants who registered with BtB did not complete the postassessment surveys. It is possible that their responses to the CSQ would have suggested that they did not find the intervention acceptable.

### Future Research and Conclusions

These findings suggest that further studies on cCBT interventions for treating depressive symptoms in veterans are warranted. Furthermore, the results suggest that unguided implementation of such interventions (ie, those that do not include regular interactions with a health care provider) may be a viable treatment delivery modality, at least for a significant proportion of veterans. Future research is needed to further develop cCBT interventions for veterans. Specifically, research is needed to establish the relative efficacy of cCBTs compared with standard face-to-face therapy among veterans, as well as with other treatment modalities that improve access to evidence-based psychotherapy for depression (eg, group-therapy delivery formats). Future research could also investigate the differences between veterans who may benefit from cCBTs on their own and those for whom additional support (eg, contact with peer support specialists) could help with successful therapy completion [58]. Finally, future research is required to empirically establish which veterans are most likely to benefit from cCBT interventions and which require other treatment modalities.

## Appendix

Multimedia Appendix 1 Measures and administration and comparison of participants completing the postintervention survey versus not completing postintervention survey.

## Abbreviations

BtB: Beating the Blues
CBT: cognitive behavioral therapy
cCBT: computerized cognitive behavioral therapies
CSQ: Client Satisfaction Questionnaire
EMR: electronic medical record
GAD-7: Generalized Anxiety Disorder Inventory-7
IEUQ: Internet Evaluation and Utility Questionnaire
IIEQ: Internet Impact and Effectiveness Questionnaire
PC-MHI: Primary Care and Mental Health Integration
PHQ-9: Patient Health Questionnaire-9
REDCap: Research Electronic Data Capture
RFT: reasons for termination
VA: Veterans Affairs
VAMC: Veterans Affairs Medical Center
