# Supplementary material for: Computerized Cognitive Behavioral Therapy Intervention for Depression Among Veterans: Acceptability and Feasibility Study
Source: JMIR Form Res. 2022 Apr 25;6(4):e31835. doi: 10.2196/31835 (PMC9086870; doi:10.2196/31835)
Supplement: Multimedia Appendix 1 [file formative_v6i4e31835_app1.docx]

Table S1. Measures and Administration

| Measure | Pre-Intervention Assessment | Post-Intervention Assessment |
| --- | --- | --- |
| Client Satisfaction Questionnaire |  | X |
| Demographics Questionnaire | X |  |
| Generalized Anxiety Disorder-7 Inventory | X | X |
| Internet Evaluation and Utility Questionnaire |  | X |
| Internet Impact and Effectiveness Questionnaire |  | X |
| Patient Health Questionnaire-9 | X | X |
| Reasons for Termination- Adapted for written responses |  | X (for those completing 1-7 modules) |

Table S2. Comparison of Participants Completing the Post-Intervention Survey vs Not Completing Post-Intervention Survey

| Variable | Participants Completing All Post-Assessment Surveys | Participants Not Completing All Post-Assessment Surveys | P value |
| --- | --- | --- | --- |
| N | 29 | 14 |  |
| Age, Mean ± SD (range) | 58.7 ± 8.6 (41-74) | 54.2 ± 12.1 (35-73) | .37 |
| Self-Identified Gender, N (%) |  |  | .37 |
| Male | 26 (90%) | 11 (79%) |  |
| Female | 3 (10%) | 3 (21%) |  |
| Racial Background, N (%) |  |  | .29 |
| Caucasian/White | 22 (76%) | 12 (86%) |  |
| Black or African American | 4 (14%) | 1 (7%) |  |
| Native American/Alaskan Native | 3 (10%) | 0 (0%) |  |
| Asian | 0 (0%) | 1 (7%) |  |
| Other | 0 (0%) | 0 (0%) |  |
| Ethnicity, N (%) |  |  | .43 |
| Hispanic/Latino(a) | 4 (14%) | 2 (14%) |  |
| Non-Hispanic/Latino(a) | 25 (86%) | 11 (79%) |  |
| Refused to Respond | 0 (0%) | 1 (7%) |  |
| Highest Level of Education, N (%) |  |  | .81 |
| High school diploma or equivalent | 1 (3%) | 2 (14%) |  |
| Some college, no degree | 10 (34%) | 5 (36%) |  |
| Associate’s degree | 4 (14%) | 2 (33%) |  |
| Bachelor’s degree | 6 (21%) | 3 (21%) |  |
| Master’s degree | 6 (21%) | 1 (7%) |  |
| Doctoral degree | 2 (7%) | 1 (7%) |  |
| Marital/Relationship Status, N (%) |  |  | .18 |
| Married | 16 (55%) | 6 (43%) |  |
| Single | 8 (28%) | 2 (14%) |  |
| Cohabitating | 1 (3%) | 0 (0%) |  |
| Divorced/Separated | 4 (14%) | 6 (43%) |  |
| Sexual Orientation, N (%) |  |  | -- |
| Heterosexual | 29 (100%) | 14 (100%) |  |
| Employment Status, N (%)* |  |  | .91 |
| Employed Full-Time | 8 (28%) | 4 (29%) |  |
| Employed Part-Time | 2 (7%) | 2 (14%) |  |
| Unemployed, not currently seeking employment | 1 (3%) | 0 (0%) |  |
| Unemployed, seeking employment | 4 (14%) | 3 (21%) |  |
| Retired | 13 (45%) | 5 (36%) |  |
| Current student, N (%) | 0 (0%) | 1 (7%) | .33 |
| Currently homeless, N (%) | 0 (0%) | 0 (0%) | -- |
| Ever homeless, N (%) | 11 (38%) | 4 (29%) | .74 |
| Branch of Military Service, N (%) |  |  | .52 |
| Army AD/NG/RSV | 11 (38%) | 5 (36%) |  |
| Air Force AD/NG/RSV | 10 (34%) | 2 (14%) |  |
| Navy AD/RSV | 4 (14%) | 3 (21%) |  |
| Other AD/RSV | 3 (10%) | 3 (21%) |  |
| Multiple Branches | 1 (3%) | 1 (7%) |  |
| Total Months Active Duty Service, Mean (SD) | 85 (79) | 52.2 (45) | .09 |
| Total Months Reserve Service, Mean (SD) | 26 (61) | 30.1 (51) | .55 |
| Service Era, N (%) |  |  | .42 |
| Vietnam (Aug 1964 – May 1975) | 7 (24%) | 4 (29%) |  |
| Post-Vietnam/Peacetime (May 1975 – July 1990) | 16 (55%) | 5 (36%) |  |
| Desert-Storm/Desert-Shield (Aug 1990 – Aug 2001) | 14 (18%) | 6 (43%) |  |
| OEF/OIF/OND (September 2001 – Present) | 6 (21%) | 6 (43%) |  |
| Other | 1 (33%) | 2 (14%) |  |
| Highest rank at separation or current rank, N (%) |  |  | .44 |
| Enlisted | 19 (67%) | 12 (86%) |  |
| Non-Commissioned Officer | 7 (24%) | 1 (7%) |  |
| Officer | 3 (10%) | 1 (7%) |  |
| Number of deployments, Median (IQR) | 1 (2.0) | 1 (2.0) | .08 |
| Number of Deployments N (%) |  |  | .23 |
| 0 | 5 (17%) | 6 (43%) |  |
| 1 | 10 (34%) | 4 (29%) |  |
| 2+ | 14 (48%) | 4 (29%) |  |
| Number of combat tours, Median (IQR) | 1 (1) | 0 (1) | .28 |
| Number of Combat Tours N (%) |  |  | .62 |
| 0 | 13 (45%) | 9 (64%) |  |
| 1 | 10 (34%) | 3 (21%) |  |
| 2+ | 6 (21%) | 2 (14%) |  |
| PHQ-9 at baseline Mean (SD) | 12.7 (5.4)* | 12.5 (3.3) | .90 |
| GAD-7 at baseline Mean (SD) | 9.3 (4.8)* | 8.5 (5.1) | .37 |

* N=28, one participant did not complete the PHQ-9 or GAD-7
